# Metabolic Syndrome and PCOS: Pathogenesis and the Role of Metabolites

**DOI:** 10.3390/metabo11120869

**Published:** 2021-12-14

**Authors:** Weixuan Chen, Yanli Pang

**Affiliations:** 1 Center for Reproductive Medicine, Department of Obstetrics and Gynecology, Peking University Third Hospital, Beijing 100191, China; weixuanchen@bjmu.edu.cn; 2 National Clinical Research Center for Obstetrics and Gynecology (Peking University Third Hospital), Beijing 100191, China; 3 Key Laboratory of Assisted Reproduction (Peking University), Ministry of Education, Beijing 100191, China; 4 Beijing Key Laboratory of Reproductive Endocrinology and Assisted Reproductive Technology, Beijing 100191, China; 5 Research Units of Comprehensive Diagnosis and Treatment of Oocyte Maturation Arrest, Chinese Academy of Medical Sciences, Beijing 100191, China

**Keywords:** PCOS, metabolic syndrome, metabolites, gut microbiota, clinical application

## Abstract

Polycystic ovary syndrome (PCOS) is one of the most common endocrine diseases among women of reproductive age and is associated with many metabolic manifestations, such as obesity, insulin resistance (IR) and hyperandrogenism. The underlying pathogenesis of these metabolic symptoms has not yet been fully elucidated. With the application of metabolomics techniques, a variety of metabolite changes have been observed in the serum and follicular fluid (FF) of PCOS patients and animal models. Changes in metabolites result from the daily diet and occur during uncommon physiological routines. However, some of these metabolite changes may provide evidence to explain possible mechanisms and new approaches for prevention and therapy. This article reviews the pathogenesis of PCOS metabolic symptoms and the relationship between metabolites and the pathophysiology of PCOS. Furthermore, the potential clinical application of some specific metabolites will be discussed.

## 1. Introduction

Polycystic ovary syndrome (PCOS) is one of the most complicated and heterogeneous endocrine disorders, with a prevalence ranging from approximately 6% (applying the older diagnostic criteria: National Institutes of Health Consensus 1990) to 20% (according to the current most commonly used criteria: the Rotterdam 2003) in women of reproductive age [1,2,3]. There are three criteria included in actual diagnostic criteria, including the Rotterdam 2003, the Androgen Excess and PCOS Society 2006 and National Institutes of Health Consensus 2012. Among these criteria, the Rotterdam criteria are the most extensive and widely used [4]. According to these criteria, three characteristics are proposed: (1) clinical or biochemical hyperandrogenism or both, (2) oligo-anovulation, and (3) polycystic ovary morphology (PCOM) (ultrasonography indicating the presence of ≥12 follicles with a maximum diameter of 2–9 mm or any ovarian volume >10 mL). A woman with PCOS must meet at least two of the three characteristics, and other causes of hyperandrogenism, such as nonclassical congenital adrenal hyperplasia and hyperprolactinemia, must be ruled out [5]. According to these diagnostic criteria, PCOS is divided into four phenotypes according to severity [6,7] (Table 1). Although there are many versions of PCOS diagnostic criteria, the etiology of PCOS remains obscure. This may be explained by multiple factors, including genetics, environment, and lifestyle [8]. PCOS also shows heterogeneity in regard to metabolic disorders [1]. This background indicates that the daily lifestyle and diet as well as metabolites generated may have a substantial influence on the pathogenesis of PCOS. Consequently, the number of clinical and basic studies on metabolic manifestations and metabolites of PCOS has increased rapidly. In this review, we aim to summarize the metabolic symptoms of PCOS, explore the pathogenesis of metabolic disorders, and perform a comprehensive review on the role of metabolites in the onset and development of metabolic features of PCOS.

## 2. Metabolic Dysfunction in PCOS

The clinical features of PCOS, such as insulin resistance, obesity, dyslipidaemia and hyperandrogenism, can be classified as metabolic syndrome. Accordingly, 43% of adult women and nearly one-third of adolescent teenagers with PCOS have metabolic syndrome [9].

### 2.1. Insulin Resistance in PCOS

Insulin resistance (IR) is common in PCOS patients. IR has been reported in approximately 50–80% of women with different phenotypes of PCOS in different races [10,11,12]. Compensatory hyperinsulinism could also exist in many PCOS patients on account of low insulin sensitivity in peripheral tissues of skeletal muscle and adipose tissue and the abnormality of insulin receptors [13]. The main mechanism of insulin receptor abnormality leading to IR is the post-binding defect due to excessive serine phosphorylation and decreased tyrosine phosphorylation, which decrease insulin activation of the phosphatidylinositol-3-kinase (PI3k) signaling pathway that activates glucose transport [14]. In recent years, there are some new information about IR in PCOS. For example, the presence of microRNA alterations in PCOS has been confirmed by many studies, but the mechanism is unknown. Dong et al. have shown that one of microRNA: miR-122 may lead to IR by inhibiting the expression of insulin-like growth factor 1, which provides a new idea on the mechanism of IR in PCOS [15]. In addition, Zhang et al. recently discovered that there is a relationship between IR and autophagy. They clarified that high mobility group box 1, a damage-associated molecular pattern molecule, can contribute to IR in granulosa cells by exacerbating autophagy [16]. And it is well known that intestinal flora is disturbed in PCOS (we will discuss later), dysbiosis in PCOS may also participate in IR by some potential mechanisms such as endotoxemia, some gut-brain peptides, hyperandrogenism and some abnormal metabolites [17]. Lastly, mitochondrial dysfunction, endoplasmic reticulum stress (ER stress) and oxidative stress were also found to play a role in IR through electroacupuncture therapy [18,19].

### 2.2. Obesity in PCOS

Obesity, especially abdominal obesity, is a common manifestation of PCOS, and the prevalence depends on geographic location and ethnicity [20]. Studies have shown that abdominal obesity may be associated with a variety of clinical features of PCOS. For example, due to adipose tissue dysfunction, adipocytes secrete non-physiological levels of adipokines, including IL6, IL8, TNF-α, leptin, adiponectin, resistin, lipocalin 2, monocyte chemoattractant protein-1 (MCP1), retinol binding protein-4 (RBP4), and CXC-chemokine ligand 5 (CXCL5), which may be involved in IR [21,22,23,24]. In addition, a recent study has indicated that obesity may function as a better predictor of skeletal muscle mass in PCOS women than hyperandrogenism and IR, which may aggravate PCOS complications [25]. Interestingly, adipose tissue dysfunction can affect follicular development. A recent study showed that IL-10 secreted by adipocytes tampers with VEGF-induced angiogenesis and further disrupts folliculogenesis [26]. Moreover, molecular mechanisms about androgens and adipose function in PCOS were mentioned recently. Lerner et al. revealed that excess androgen can inhibit brown adipogenesis, attenuating the activation of thermogenesis and reducing mitochondrial respiration in brown adipose tissue [27]. Zhou et al. used bioinformatics analysis to identify CHRDL1 gene which may be responsible for obesity of PCOS by inhibiting bone morphogenetic protein 4 signaling or regulating IGF-1 [28].

### 2.3. Hyperandrogenism in PCOS

One of the PCOS diagnosis criteria is hyperandrogenism. IR, obesity and hyperandrogenism are inseparable in the pathogenesis of PCOS. Hyperinsulinaemia caused by IR exerts a cogonadotropin effect on the ovaries and decreases the expression of sex hormone-binding protein (SHBG), leading to the onset of hyperandrogenism [29,30]. Androgens can induce the accumulation of adipose tissue, especially abdominal fat tissue, and cause IR in subcutaneous adipose tissue [31,32]. In humans, androgen plays a dual role in folliculogenesis: a low dose of androgens promotes follicle growth, while a high level of androgens could augment the secretion of anti-Müllerian hormone (AMH) in granulosa cells, thus inhibiting follicular development [33]. Several studies have also reported other potential mechanisms of hyperandrogenism-induced PCOS, such as dihydrotestosterone (DHT), which could contribute to mitochondrial fission in granulosa cells of PCOS patients, and excess androgens induce ER stress, which may damage oocyte quality [34,35]. Besides, Wang et al. found that hyperandrogenism may contribute to chronic low-grade inflammation in ovary and granulosa cells of PCOS by generating NLRP3 inflammasome, which further promotes granulosa cells pyroptotic death and ovarian fibrosis [36]. Therefore, hyperandrogenism plays a complicated role in PCOS.

### 2.4. Dyslipidaemia in PCOS

Dyslipidaemia is regarded as an important metabolic phenotype, although it is not a diagnostic criterion. It has been reported that the prevalence of dyslipidaemia in PCOS patients is 70%, and the levels of low-density lipoprotein cholesterol (LDL-c), very-low-density lipoprotein cholesterol (VLDL-c), triglycerides (Tgs), and free fatty acid are increased, while the levels of high-density lipoprotein cholesterol (HDL-c) are decreased [37,38]. Moreover, it seems that nonobese patients have a higher prevalence of hypertriglyceridemia and low HDL [39]. And there is evidence suggesting that black women with PCOS have lower Tgs than white women, although the risk of cardiometabolic disease is higher [40]. Dyslipidaemia were also reported to affect long-term outcomes of PCOS patients. Wekker et al. revealed that PCOS women had a more adverse lipid profile and had a higher risk for non-fatal cerebrovascular disease events [41].

### 2.5. Other Metabolic Consequences in PCOS

#### 2.5.1. Nonalcoholic Fatty Liver Disease (NAFLD) and Nonalcoholic Steatohepatitis (NASH)

Many metabolic manifestations, such as IR, hyperandrogenism and dyslipidaemia, in women with PCOS are similar to the metabolic manifestations of NAFLD and NASH. Additionally, many studies have confirmed a high prevalence of NAFLD in women with PCOS [42]. Additionally, the fact that high androgen levels are involved in the development of hepatic steatosis in women with PCOS is widely accepted [43]. In 2020, Li et al. have demonstrated that elevated endogenous testosterone induced by letrozole can result in hepatic steatosis in PCOS rats and they further found that hyperandrogenism inhibit the AMP-activated protein kinase alpha (AMPKa) signaling, which regulates lipid metabolism, in letrozole-treated livers and dihydrotestosterone (DHT)-treated HepG2 cells [44]. Additionally, recent studies on mitochondrial dysfunction have also implied a mechanism between PCOS and NAFLD [45,46]. Due to mitochondrial gene mutations like, persistent oxidative stress (OS) from abnormal mitochondrial may worsen hyperandrogenism, IR and lipid accumulation which contribute to NAFLD and PCOS [47]. However, the specific mechanism of NAFLD in PCOS patients remains to be clarified.

#### 2.5.2. Cardiovascular Disease in PCOS

The metabolic characteristics of PCOS can lead to a variety of cardiovascular diseases (CVDs), such as hypertension, atherosclerosis, and coronary heart disease. An increased risk of CVD is demonstrated by surrogate markers such as flow-mediated dilation, carotid intima-media thickness and coronary artery calcification [48,49,50]. Accordingly, mitochondrial dysfunction may also play a role in CVDs of PCOS women, as cardiocytes needs much energy produced from mitochondria [51]. Apart from the influence of IR, obesity and dyslipidemia, excess androgen has been reported to lead to CVDs. Hyperandrogenism may activate the sympathetic nervous system by melanocortin-4 receptor, 20-hydroxyeicosatetraenoic acid and oxidative stress [52].However, whether these patients ultimately have a high risk of CVD is still unclear, as more detailed, larger and prospective cohort studies are still needed [53].

### 2.6. Summary of Metabolic Symptoms in PCOS

The metabolic symptoms of PCOS seem to be connected. It has been proposed that androgen excess is the beginning of a vicious cycle of metabolic disorders in PCOS patients. It is believed that with the induction of IR and hyperinsulinaemia, hyperandrogenaemia facilitates the formulation of visceral adipose tissue, which exacerbates the secretion of androgen in the ovaries and adrenal glands. Accordingly, the vicious cycle is the potential mechanism of steroidogenesis defects, and the severity depends on different factors [1,54].

## 3. Metabolites That Contribute to the Development of PCOS

In regard to the role of metabolites in PCOS, the gut microbiota is inevitably mentioned. Approximately 10^14^ microorganisms live in the human intestine, primarily those belonging to the phyla Firmicutes and Bacteroidetes, which have been found to produce potential metabolites via analytical techniques in metabolomics and interact with the human reproductive system [55,56]. Correspondingly, several studies have demonstrated that PCOS patients have gut microbiota dysbiosis and abnormal composition of metabolites, such as bile acids (BAs), short-chain fatty acids (SCFAs), branched-chain amino acids (BCAAs), ceramides, trimethylamine N-oxide (TMAO). BAs are cholesterol-derived in humans and can be re-metabolized by intestinal bacteria and ceramides can be produced in a variety of tissues in the body. Besides, although BCAAs, SCFA and TMAO cannot be synthesized by the human body, they can be produced from food and interact with intestinal bacteria in the body [57,58,59,60,61].

### 3.1. Microbiota Dysbiosis of PCOS

From the time the concept of the existence of dysbiosis of gut microbiota in PCOS was introduced to the present, there are various studies exploring the potential mechanism. Kelly et al. found that hyperandrogenaemia in letrozole-induced PCOS mouse may significantly alter the gut microbiome independently of diet [62]. Subsequently, other teams have discovered that dysbiosis in intestinal flora exist in both mouse models of PCOS and in patients with PCOS. Specifically, Torres et al. revealed that hyperandrogenism is strongly associated with the biodiversity of microbiome as with α and β diversity [63]. Lindheim et al. further revealed three bacterial taxa are lower abundance in PCOS, including phylum Tenericutes, the order ML615J-28 (phylum Tenericutes) and the family S24-7 (phylum Bacteroidetes) [64]. Liu et al. elaborated that Bacteroides, Escherichia/Shigella, Streptococcus and Akkermansia are negatively correlated with ghrelin and Bacteroides, Escherichia/Shigella, Streptococcus are positively correlated with metabolic parameters and testosterone. Additionally, the decrease in Akkermansia and increase in lipopolysaccharide (LPS) -producing bacteria are also discovered in their study [65]. Similarly, Chu et al. used metagenomic species analysis revealed some strains such as Parabacteroides merdae, Bacteroides fragilis, Escherichia and Shigella are enriched. They further analyzed some abundant strains and put forward some potential mechanism between dysbiosis of microbiome and PCOS: flora may harm intestinal gut permeability and cause barrier dysfunction in intestinal tract, some bacteria may produce much reactive oxygen species, enriched Gram-negative bacteria can produce LPS [66]. In addition, some differences in the composition of gut bacteria have been reported across weight levels and the presence or absence of IR. For example, Mammadova et al. suggested that gut microbiome of lean PCOS patients is similar with controls in bacterial richness and diversity [67]. However, Chen et al. indicated that there are not significant differences between normal BMI PCOS and high BMI PCOS patients in bacterial diversity and community and they found the abnormal gut bacteria in PCOS maybe due to the bacterial response to stress which in turn leads to reduced stress-associated FK506binding protein 5 DNA methylation [68]. Accordingly, the abundance of bacteria in PCOS with IR are different from PCOS-alone and healthy patients [69]. Moreover, some studies reported that prenatal androgen exposure may cause gut microbial dysbiosis and it may lead to PCOS in later life [70,71].

### 3.2. Bile Acids

Primary bile acids (BAs), including chenodeoxycholic acid (CDCA) and cholic acid, are produced from cholesterol in the liver, are conjugated with taurine and glycine to form primary conjugated bile acids and are then transported to the intestine [72]. With enzyme-catalyzed and deconjugated reactions, primary bile acids are converted into secondary bile acids comprising lithocholic acids (LCAs) and deoxycholic acids (DCAs) during contact with the intestinal flora [73,74]. Farnesoid X receptor (FXR) and G protein coupled receptor (GPCR), especially transmembrane G coupled receptor 5 (TGR5), are the main receptors of BAs. FXR has been extensively studied in BA metabolism. There is mutual control between BAs and FXR. In detail, FXR can activate the expression of fibroblast growth factor 15 (FGF15) and FGF19 in the intestinal tracts of mice and humans, respectively, inhibiting the expression of the enzymes cytochrome P450 7A1 (CYP7A1) and cytochrome P450 8B1 (CYP8B1), which are involved in the synthesis of BAs [75]. However, the effects of FXR are not uniform in different tissues. In the liver, activation of FXR may have beneficial metabolic effects, such as ameliorating IR and high-fat diet-induced obesity, but the effect in the intestine is adverse [76].

As previously mentioned, dysbiosis of the intestinal flora in PCOS patients has been verified [57,65]. Our group found that the specific bacterium Bacteroides vulgatus was markedly elevated in women with PCOS and that the bacterium expressed the bile salt hydrolase (BSH) gene. BSH is the main enzyme that deconjugates BAs in intestinal bacteria. We further demonstrated that glycodeoxycholic acid (GDCA) and tauroursodeoxycholic acid (TUDCA) were decreased due to the deconjugation of BSH in PCOS patients and that supplementation with these bile acids can improve the PCOS phenotype by activating TGR5 and further enhancing IL-22 secretion by intestinal Group 3 innate lymphoid cells (ILC3s) [58]. Another study uncovered the potential mechanism of TGR5 and FXR, suggesting that some BAs indirectly promote glucagon-like peptide-1 (GLP-1) secretion by activating TGR5 or FXR, thereby lowering blood glucose and providing a possible therapy for PCOS [77,78]. In addition, it has been reported that the increase in circulating primary conjugated BAs is associated with hyperandrogenism, and the presence of FXR in ovarian granulosa cells and the intestine also implies that primary conjugated BAs contribute to hyperandrogenism via this mechanism [79,80].

### 3.3. Short-Chain Fatty Acids

Fatty acids with carbon chains with less than 6 carbon atoms are classified as SCFAs based on their aliphatic tail length. SCFAs are derived from dietary fiber, such as oligofructose and resistant starch, from food and are fermented by gut microbes because there are no human digestive enzymes for dietary fiber. In the caecum, colon and feces, which are primary fermentation places [81,82], the concentrations of acetate, propionate and butyrate are highest, and they play a primary role in the metabolism of SCFAs via two types of fatty acid receptors (FFARs): FFAR3 (GPR41) and FFAR2 (GPR43). These receptors are identified as G protein-coupled receptors, which are coupled to G_i/o,_ and FFAR2 is additionally coupled to G_q,_ which recruits β-arrestin-2 [83].

It has been reported that the composition of SCFAs is different between healthy controls and patients with metabolic syndrome due to changes in intestinal flora. Qin et al. and Karlsson et al. discovered that there is a higher proportion of Clostridiales and a lower concentration of butyrate in patients with T2DM [84,85]. Additionally, studies have demonstrated that the abundance of some intestinal flora components, including Bacteroidetes from the Bacteroidaceae, Porphyromonadaceae, and S24-7 families and Firmicutes from the Clostridiaceae, Erysipelotrichidae, Lachnospiraceae, Lactobacillaceae, and Ruminococcaceae families, which produce SCFAs, is different in PCOS patients [86].

Although few studies have shown the exact relationship between PCOS and SCFAs, some effects of FFAR3 and FFAR2 activation suggest that SCFAs may play a vital role in the pathogenesis of the PCOS phenotype. For example, a study revealed that activation of FFAR2 can stimulate the secretion of GLP-1 and peptide YY (PYY) [87]. Activation of FFAR3 can upregulate the mRNA expression of leptin [88]. These gut hormones can directly improve obesity and act indirectly through the gut-brain axis by crossing the blood–brain barrier, further influencing the hypothalamus to suppress appetite [89]. Additionally, a recent study also indicated that SCFAs may enhance insulin sensitivity by feeding mice fermentable fiber (inulin), and investigators discovered that SCFAs may ameliorate T1DM through IL-22 [90]. These effects of SCFAs may participate in the pathogenesis of PCOS.

### 3.4. Branched-Chain Amino Acids

BCAAs, including leucine, isoleucine and valine, are essential amino acids obtained from foods that cannot be synthesized by humans [91]. On the one hand, it is known that BCAAs play roles in anabolic effects on body weight, muscle protein synthesis and glucose homeostasis [92]. On the other hand, some studies have also suggested that BCAAs are associated with insulin resistance, obesity and even T2DM, which have similarities with the phenotypes of PCOS. These results indicate that BCAAs may be involved in the onset of PCOS or serve as biomarkers for PCOS [93,94].

However, findings regarding the effects of BCAAs on IR are inconsistent, and there are several possible mechanisms by which BCAAs contribute to IR. For example, excess BCAAs can activate mammalian target of rapamycin complex 1 (mTORC1), leading to serine phosphorylation of IRS-1 and IRS-2 [95]. Another mechanism is that some metabolites derived from abnormal metabolism of BCAAs may impair the function of islet β cell mitochondria [96]. Furthermore, BCAAs may induce the expression of proinflammatory genes that are involved in the development of IR [97].

Consequently, a study in 2012 suggested that the metabolism of amino acids, especially BCAAs, is disordered in PCOS [98]. A recent study revealed abnormal degradation of BCAAs in ovarian granulosa cells of PCOS patients [99]. Another study revealed that the levels of BCAAs in PCOS patients did not differ from those in healthy people after exercise, in contrast to different levels before exercise [100]. In addition, because some gut microbes can synthesize a certain amount of BCAAs in vivo, dysbiosis of gastrointestinal flora may contribute to PCOS through the BCAA pathway. For instance, Pedersen et al. reported that Prevotella copri and Bacteroides vulgatus can function as the main BCAA synthesizers in the human intestine. As mentioned before, Bacteroides vulgatus was also found to be highly abundant in the intestines of PCOS patients [59]. Pedersen et al. further demonstrated that Prevotella copri can induce insulin resistance, aggravate glucose intolerance and increase circulating levels of BCAAs [101].

### 3.5. Other Potential Metabolites

#### 3.5.1. Ceramides

Ceramides are produced by the condensation of palmitoyl-CoA and serine after four-step enzymatic catalysis. Specifically, palmitoyl-CoA and serine are transformed to 3-ketosphinganine by serine palmitoyltransferase, which is rapidly converted to sphinganine and combines with FAs to produce dihydroceramides. Finally, under the action of dihydroceramide desaturase, ceramides are synthesized. This is the de novo pathway for ceramide production [102]. There is a salvage pathway in which sphingosine and a variety of complexes generated from ceramides, such as sphingomyelin, can be transformed to ceramides via enzyme catalysis [103].

In addition, the connection between ceramides and IR has been verified. A study in 2019 revealed that a lack of ceramides can alleviate IR by inserting a conserved double bond into the backbone of ceramides, which transforms ceramides into dihydroceramides [104]. In fact, many studies have revealed that ceramides may inhibit insulin action by blocking different sites [105]. Broadly, ceramides can block the activation of Akt/PKB by protein phosphatase 2A and protein kinase C ζ [106,107].

Some studies have implied that dysbiosis of the gut microbiome is associated with high concentrations of ceramides that further lead to a series of metabolic disorders. Johnson et al. demonstrated that Bacteroidetes, which is the dominant phylum of the gut microbiome, can produce sphingolipids and further influence the production of ceramides in the host [60]. Kayser et al. also found that ceramides are associated with gut microbiota richness in individuals with obesity and impaired glucose metabolism [108]. In addition, ceramides are involved in IR by serving as a downstream effector molecule of intestinal FXR, and activation of hypoxia-inducible factor 2α resulting from intestinal hypoxia in obese patients can increase the level of ceramides and exacerbate IR [109,110].

Accordingly, because ceramides can result in IR and are associated with metabolic diseases, there may be a connection between PCOS and ceramides. Jiang et al. found that the concentration of ceramides was higher than that in healthy controls by using shotgun lipidomics and identified a combination of ceramide subclasses (OH_N16:0/N18:0) and (N22:0) ceramides that may become a new biomarker of PCOS [111]. Nevertheless, there are still only a few studies reporting on PCOS and ceramide. The mechanism of action of ceramides with different acyl chain lengths on PCOS remains to be studied.

#### 3.5.2. Trimethylamine N-oxide

Trimethylamine N-oxide (TMAO) originates from trimethylamine (TMA), which is generated from dietary components such as L-carnitine, choline and other choline-containing compounds in the intestine. TMA is then absorbed from the intestine into the portal vein circulation, where TMAO is produced in the liver by flavin monooxygenase 3 (FMO3) [112].

A variety of studies have revealed that TMAO is involved in many metabolic diseases, such as cardiovascular disease and T2DM [113,114]. A recent study also suggested that TMAO and its related metabolites may be associated with a higher risk of IR [115]. However, few studies have found concrete evidence in humans. In 2019, Heianza et al. provided more powerful support that diet-induced TMAO is associated with improvement of IR in obese people and that higher levels of TMAO are associated with less improvement in glycaemia and insulin sensitivity [116]. In fact, the underlying mechanism is unknown, but activation of protein kinase R-like endoplasmic reticulum kinase (PERK) by TMAO has attracted extensive attention [117].

Moreover, TMAO is considered a potential metabolite in the pathogenesis of PCOS. In a prospective study of 27 obese patients with PCOS, Eyupoglu et al. found for the first time that TMAO and its precursors are elevated in women with PCOS compared with in healthy women, which seems to indicate that TMAO is associated with hyperandrogenism in PCOS [118]. Interestingly, another study suggested that elevated plasma TMAO levels may be associated with the pathogenesis of PCOS rather than hyperandrogenism [119]. Therefore, although TMAO may be a potential biomarker for PCOS, the connection between TMAO and PCOS still needs further study.

## 4. Exploring Possible Metabolite-Related Clinical Interventions for PCOS

PCOS is a combination of metabolic and reproductive endocrine disorders, so there are a large number of changes in metabolites involved. In addition, there may also be multiple clinical therapies for PCOS from a metabolic perspective. Accumulating evidence has demonstrated that intestinal bacteria are an important part of this process.

### 4.1. Dietary Intervention

It is known that PCOS is a metabolic disease. Therefore, dietary interventions are considered convenient and safe treatments in clinical practice. A meta-analysis showed that a low carbohydrate diet (LCD) particularly long-term LCD is beneficial for PCOS [120]. Additionally, a ketogenic diet has also been mentioned for alleviate the phenotypes of PCOS such as disordered menstrual cycle and abnormal liver function [121,122]. Another study revealed that adhering to a Mediterranean diet (MD), which is rich in complex carbohydrates, fiber and monounsaturated fats, may be one of the most suitable diet plans for PCOS [123]. The potential mechanism may be illustrated by the ability of a MD to increase the abundance of Parabacteroides distasonis, Bacteroides thetaiotaomicron, Faecalibacterium prausnitzii, Bifidobacterium longum, and Bifidobacterium adolescentis [124]. Moreover, calorie restriction is as important as food composition and Shang et al. revealed that calorie-restricted diets may be the optimal choices for reducing IR and improving body composition [125]. Interestingly, even though differences in diet structure can have different effects on PCOS theoretically, studies show that weight loss is most beneficial for obese patients, regardless of the composition of the diet [126]. And other research also corroborated it that women with PCOS met the acceptable macronutrient distribution ranges for carbohydrate, fat, and protein but not for micronutrient [127]. As for this view, a recent study thought that PCOS patients consume less dietary fibre, vitamin D and vitamin E not for and have lower levels of GABA-producing bacteria, which may cause PCOS through the gut-brain axis [128]. Anyway, designing optimal dietary intervention should consider not only macronutrients but also micronutrients, as well as designing better clinical studies to confirm.

### 4.2. Application of Bacteria

Probiotics are defined as live microbial dietary supplements that shape and balance host intestinal bacteria [129]. Many functions of probiotics, including protecting the gut barrier, improving IR and regulating the immune system, have been revealed [130]. Studies in Iran revealed that a 12-week probiotics supplementation (Lactobacillus acidophilus, Lactobacillus casei and Bifidobacterium bifidum) can bring good metabolic effect to PCOS [131,132]. Other RCTs in Iran used a 8-week probiotics treatment, including Lactobacillus casei, Lactobacillus acidophilus, Lactobacillus rhamnosus, Lactobacillus bulgaricus, Bifidobacterium breve, Bifidobacterium longum and Streptococcus thermophiles, which also showed Homeostasis Model Assessment of Insulin Resistance (HOMA-IR) and fasting plasma glucose (FPS) are improved [133]. Meta-analysis in 2019 and 2021 illustrated that probiotics or synbiotics can improve metabolic indices such as high-density lipoprotein (HDL), triglycerides, and fasting insulin but have no obvious effect on weight, BMI, HOMA-IR and WC (waist circumference) or some inflammatory indices such as C-reactive protein (CRP) and high-sensitivity C-reactive protein (hsCRP) [134,135]. However, a meta-analysis in 2020 suggested that intake of probiotics or synbiotics can improve not only metabolic indices such as FPS, HOMA-IR and triglycerides but also hormonal and inflammatory factors such as serum testosterone, hsCRP, nitric oxide and total antioxidant capacity but not HDL [136]. In addition, the combination of probiotics with other substances such as selenium and vitamin D for the treatment of PCOS has also been reported to be beneficial [137,138]. In any case, even if some results of the meta-analysis are different, the overall effect of probiotics on PCOS from published studies is favorable. In terms of potential mechanism of probiotics, Zhang et al. reported that beneficial microbes Bifidobacterium lactis V9 can increase the growth of SCFA-producing microbes such as Akkermansia, Butyricimonas, and Faecalibacterium prausnitzii. Therefore, they concluded that the probiotic Bifidobacterium lactis V9 may improve PCOS by affecting the production of SCFAs, which in turn affects the secretion of gut-brain mediators, including ghrelin and PYY [139]. Furthermore, the mechanism by which probiotic lactic acid bacteria can improve PCOS by regulating sex hormone-related gut microbiota has been demonstrated [140].

Prebiotics are nondigestible compounds but are beneficial to the body because they are metabolized by microorganisms in the gut to selectively stimulate the growth and/or activity of gut microbiota [141]. A clinical trial in 2019 reported that the consumption of resistant dextrin as a prebiotic resulted in positive effects of reducing FPS, hsCRP, total cholesterol, triglycerides, LDL cholesterol, dehydroepiandrosterone sulfate (DHEA-S) and free testosterone and increasing HDL cholesterol in women with PCOS [142]. The potential mechanism for this effect may be enhanced production of GLP-1 and SCFAs by certain flora. However, there are few studies on the effect of prebiotics on intestinal flora. Overall, probiotics, prebiotics and synbiotics have been proven to be effective treatments for PCOS [135].

Last, fecal microbiota transplantation (FMT) is also a novel and effective means of treating metabolic diseases and can also be used for PCOS. The essence of FMT is the transfer of intestinal microorganisms from the donor to the recipient [143]. Guo et al. first reported that the estrous cycles and ovarian morphologies of PCOS rats were improved by treatment with Lactobacillus and FMT from healthy rats [57]. Additionally, Qi et al. reconfirmed that transplantation of intestinal flora from PCOS mice enables normal mice to exhibit the phenotypes of PCOS, and they further identified a specific bacterium from PCOS mice: Bacteroides vulgatus [58]. There are several studies about FMT and other metabolic diseases but few studies on FMT and PCOS.

### 4.3. Vitamin D

In recent years, there has been an increasing number of studies on vitamin D and PCOS. It has been shown that there is a relationship between vitamin D deficiency and the development of PCOS [144]. Therefore, vitamin D has been explored for the treatment of PCOS. Several studies have reported that vitamin D treatment can reduce AMH levels in PCOS patients and improve IR and embryo quality from in vitro fertilization [145,146]. Furthermore, an RCT revealed that vitamin D3 at a dose of 50,000 IU per week can reduce hirsutism scores and androgen levels in obese PCOS patients [147]. However, the underlying mechanism has not been clearly explained, and because different doses of vitamin D may have different effects, sound research is still needed to find the right dose.

### 4.4. Inositol

Apart from vitamin D, inositol, which is considered a B vitamin, can also play a role in treating PCOS. Inositol exists in two main forms: myo-inositol (MI) and D-chiro-inositol (DCI). MI can be converted to DCI under insulin stimulation [148]. Because MI and DCI can act as insulin sensitizers to restore IR and improve the oocyte quality, there are many research exploring the right dosage and isoforms of inositol and the right ratio of MI to DCI for PCOS has been widely studied [149,150]. Many studies have compared the effects of different combination types of MI alone, MI with DCI, and MI combined with metformin on the treatment of PCOS [151,152,153]. They found that both MI and DCI can improve the metabolic profile of PCOS, specifically, MI shows better metabolic improvement and DCI reduced hyperandrogenism better and MI may be more effective than metformin in terms of adverse effects [149,154]. MI and DCI (40:1) have been identified as effective treatments for PCOS and the dosage of DCI should less than 300 mg to protect oocyte quality [155,156]. Additionally, a study reported that the use of alpha-lactalbumin in combination with MI increased the absorption of MI, which may provide a more effective treatment for PCOS [157].

### 4.5. GLP-1RA

GLP-1 receptor agonists (GLP-1RA) such as liraglutide has emerged as a new treatment for PCOS in recent years as GLP-1 has many unique advantages for the treatment of metabolic diseases such as inhibition of gastric emptying and food intake and augmentation of insulin secretion [158]. Many clinical research has shown that GLP-1RA has good metabolic effects for weight loss, decreasing androgen level, improving IR [159]. And some studies have reported reproductive benefits from GLP-1RA, specifically, administration of GLP-1RA increase in vitro fertilization pregnancy rates and natural pregnancy rate in obese PCOS patients [160,161].

### 4.6. Others

Statins are one of most widely used drugs for dyslipidaemia, which have been reported to lower testosterone in PCOS patients. For example, a meta-analysis in 2021 showed atorvastatin can lower the total testosterone, free androgen index, androstenedione, and DHEAS in PCOS women [162]. And simvastatin combined with metformin show a better ability of reducing total testosterone, LH:FSH ratio and LDL cholesterol [163].

A substance we get daily from red meat: L-carnitine, has been reported to improve endocrine function and folliculogenesis by reducing inflammation, oxidative stress and apoptosis in PCOS mice [164]. And a randomized clinical trial in 2021 also suggested that 12-week L-carnitine supplementation can improve IR in obese PCOS patients [165].

Photobiomodulation for treating PCOS is a new filed. Naseri et al. indicated that near-infrared laser can increase ovarian activity to produce oocyte [166]. Besides, treatment of EV-induced PCOS rats with 808 nm laser reduces the number of follicular cysts and increases the number of follicles [167]. Although there are scarce clinical data on this area, still it deserves further exploration.

In addition, some cytokines, such as IL-22, some endogenous substances such as microRNA, atrial natriuretic peptide and apelin, a newly identified adipokine have been proposed as novel therapeutic targets for the treatment of PCOS [58,168,169,170].

## 5. Conclusions

**Figure 1:**
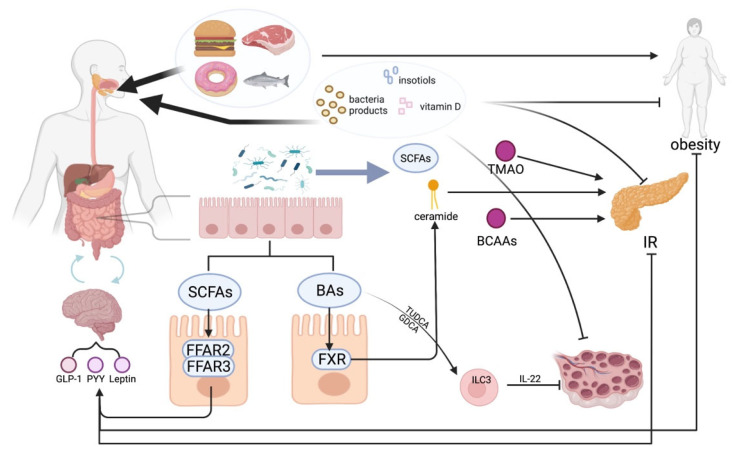
The role of some metabolites in the pathogenesis of PCOS. SCFAs: short-chain fatty acids, TMAO: Trimethylamine N-oxide, BCAAs: branched-chain amino acids, IR: insulin resistance, FFAR: fatty acid receptor, GLP-1: glucagon-like peptide-1, PYY: peptide YY, FXR: Farnesoid X receptor, TUDCA: tauroursodeoxycholic acid, GDCA: glycodeoxycholic acid, ILC3: intestinal Group 3 innate lymphoid cell.

With a large number of metabolomic studies, an increasing number of abnormal metabolites are being identified in PCOS patients. How these abnormal metabolites are produced and how they are involved in the development of PCOS still require a great deal of research. It is worth noting that intestinal flora may play an important role in this process. In this review, we systematically describe the various metabolic phenotypes of PCOS and the relationship between disordered metabolites and intestinal flora and further summarize the more promising therapeutic approaches for PCOS. However, only a few characteristic metabolites are presented here, and some of them cannot explain all phenotypes of PCOS. Thus, these questions may indicate that there are many metabolites that act together to cause PCOS. Apart from that, further research on intestinal flora in the metabolism of PCOS patients is still needed, especially in identifying specific species and the specific pathogenic mechanisms in PCOS. Finally, due to the complex metabolic nature of PCOS, therapies that can regulate metabolism, such as modulation of the host gut bacteria or supplementation of deficient metabolites in the body, may be developed as more effective treatments in the future. However, there are few studies on metabolites or FMT in the context of clinical translational medicine. As a result, it is necessary to design better clinical studies for testing these potential treatments (Figure 1).

## Figures and Tables

**Table 1:** The four phenotypes of PCOS according to the 2003 Rotterdam Criteria [4].

Phenotype 1	Phenotype 2	Phenotype 3	Phenotype 4
Oligo-anovulation	Androgen excess	Androgen excess	Androgen excess
PCOM	PCOM	Oligo-anovulation	Oligo-anovulation
			PCOM

Modified from group, T.R.E.A.s.P.c.w. Revised 2003 consensus on diagnostic criteria and long-term health risks related to polycystic ovary syndrome (PCOS). Hum. Reprod. 2004, 19, 41–47.
